# Physical Activity Modifies the Metabolic Profile of CD4 ^+^ and CD8 ^+^ T‐Cell Subtypes at Rest and Upon Activation in Older Adults

**DOI:** 10.1111/acel.70104

**Published:** 2025-05-21

**Authors:** Jon Hazeldine, Edward Withnall, Alba Llibre, Niharika A. Duggal, Janet M. Lord, Amanda V. Sardeli

**Affiliations:** ^1^ Department of Inflammation and Aging University of Birmingham Birmingham UK

**Keywords:** aging, cellular immunology, cytokines, inflammation, mitochondria, senescence, T cell, training

## Abstract

T‐cell metabolism is a key regulator of immune function. Metabolic dysfunction in T cells from young mice results in an aged phenotype, accelerating immunosenescence. Physical activity (PA) maintains T‐cell function and delays immunosenescence in older adults, but the underlying mechanisms are poorly understood. We investigated the effects of PA on the metabolic and functional profiles at a single‐cell resolution of resting and stimulated T cells from young adults (*N* = 9, 23 ± 3 years) and physically active older adults clustered between higher PA (HPA, *N* = 9, 75.5 ± 4.7 years) or lower PA levels (LPA, *N* = 10, 76.4 ± 2.1 years). Compared to young donors, HPA older adults had higher mitochondrial dependence (MD) and lower glucose dependence (GD) in unstimulated naïve, central memory (CM) and effector memory (EM) CD4^+^ and EM CD8^+^ T cells, while LPA older adults had higher overall protein synthesis in naïve and EM CD4^+^ and CD8^+^. In response to PMA and Ionomycin stimulation, there was a similar increase in GD and a reduction in MD across groups for most T‐cell subsets. Although LPA and HPA underwent a higher increase in protein synthesis upon activation compared to the young subjects, HPA did not exhibit the excessive increase in the percentage of IL‐6^+^ T cells observed in the LPA group compared to young subjects. Taken together, our data provide evidence of a higher energy demand, impaired metabolic flexibility, and hyperinflammatory responses in aged T cells, and PA reduces metabolic demand in these cells, potentially through increased MD and improved metabolic flexibility.

## Graphical abstract

The figure combines the effects of aging and physical activity on different T‐cell subsets. Overall, our data provide evidence of higher energy demand, impaired metabolic flexibility, and hyperinflammatory responses in aged T cells, and PA reduces metabolic demand in these cells, potentially through increased MD and improved metabolic flexibility.

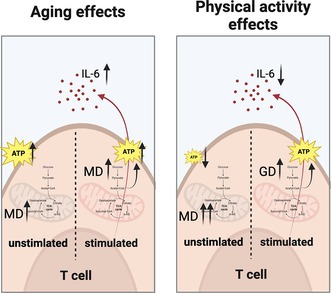

**Abbreviations**
CM: Central memory
EM: Effector memory
EMRA: Effector memory re‐expressing CD45RA
GD: Glucose dependence
HPA: High physical activity‐level group
IFNγ: Interferon gamma
IL‐17: Interleukin 17
IL‐6: Interleukin 6
LPA: Low physical activity‐level group
MD: Mitochondrial dependence
mtDNA: Mitochondrial DNA
MVPA: Moderate to vigorous physical activity
PA: Physical activity
ROS: Reactive oxygen species
TNF‐α: Tumor necrosis factor alpha

## 1 Introduction, Results, and Discussion

Aging is associated with a progressive functional decline in the immune system (immunosenescence). Hallmarks of T‐cell aging include reduced naïve T‐cell counts, accumulation of terminally differentiated memory cells with senescent properties (highly inflammatory) and a state of functional decline that ultimately leads to an increased susceptibility to disease and impaired response to vaccination and infections (Desdín‐Micó et al. 2020; Møller et al. 2022; Pinti et al. 2016; Liu et al. 2023). Physical activity (PA) benefits aged T cells, which is reflected in clinical outcomes such as better vaccination responses (Pascoe et al. 2014), reduced severity and duration of respiratory infections (Grande et al. 2020) and reduced age‐related multimorbidity (Duggal et al. 2019; Sallis et al. 2021). Previously, we have shown that maintenance of PA into older age can partially prevent immunosenescence, with PA preserving thymic output and reducing systemic inflammation (Duggal et al. 2018). However, the mechanisms by which PA preserves a more youthful immune phenotype are poorly understood.

Effective T‐cell function depends on the activation of specific metabolic pathways (Voss et al. 2021), which are known to be altered with aging (Møller et al. 2022; Quinn et al. 2023). For example, effector T cells rapidly upregulate glycolytic metabolism (glycolytic shift) (Møller et al. 2022; Quinn et al. 2023) to generate biosynthetic intermediates essential for proliferation. Inhibition of glycolysis in activated T cells impedes effector cell differentiation, shifting cells toward memory subtypes that display increased oxidative metabolism and augmented mitochondrial fusion to sustain cellular longevity (Møller et al. 2022; Quinn et al. 2023; Sukumar et al. 2013). PA is a potent metabolic regulator (Hodgman et al. 2023; Smith et al. 2023), and there is evidence that it can improve mitochondrial respiration in T cells (Andonian et al. 2020; Gebhardt et al. 2024). We therefore hypothesized that PA maintains T‐cell function in older adults by ameliorating metabolic dysfunction.

We recruited 9 young (23 ± 3 years; 7 males, 2 females) and 19 healthy older adults, subdivided based on their PA levels, as either higher PA (HPA, moderate to vigorous [MVPA] 147 ± 18 min/day; 76 ± 4 years; 4 males, 5 females) or lower PA (LPA, MVPA 74 ± 27 min/day; 72 ± 2 years; 4 males, 6 females). PA levels were determined by accelerometry (ActiGraph GT9X‐BT, ActiGraph LLC, Pensacola, FL, USA), by the average of seven‐day recordings. Both groups had an average MVPA above the minimum recommendations of 150 min of PA per week (Garber et al. 2011), meaning that even the LPA within this cohort are physically active. Peripheral blood mononuclear cells (PBMCs) were isolated, and the metabolic profiles of helper CD4^+^ and cytotoxic CD8^+^ T‐cell subtypes at rest and postactivation (PMA [50 ng/mL] and Ionomycin [500 ng/mL]) were examined by flow cytometry (Argüello et al. 2020).

Using SCENITH (Argüello et al. 2020), a flow cytometry‐based method for metabolic profiling, we assessed glucose dependence (GD) and mitochondrial dependence (MD) as a percentage of total protein synthesis. Recognizing the heterogeneity within T cells, we studied the metabolic profiles of naive (CCR7^+^ CD45RA^+^), central memory (CM, CCR7^+^ CD45RA^‐^), effector memory (EM, CCR7^−^ CD45RA^−^) and terminally differentiated effector memory (EMRA, CCR7^−^ CD45RA^+^) CD4^+^ and CD8^+^ T cells, both at rest (unstimulated) and following activation. The Supporting Information provides detailed methods (S1), gating strategies (S2 and S3), cohort characteristics (S4) and key materials used for the flow cytometry experiments (S5).

### 1.1 Metabolic Profile of Unstimulated CD4 ^+^ and CD8 ^+^ T Cell

**FIGURE 1:**
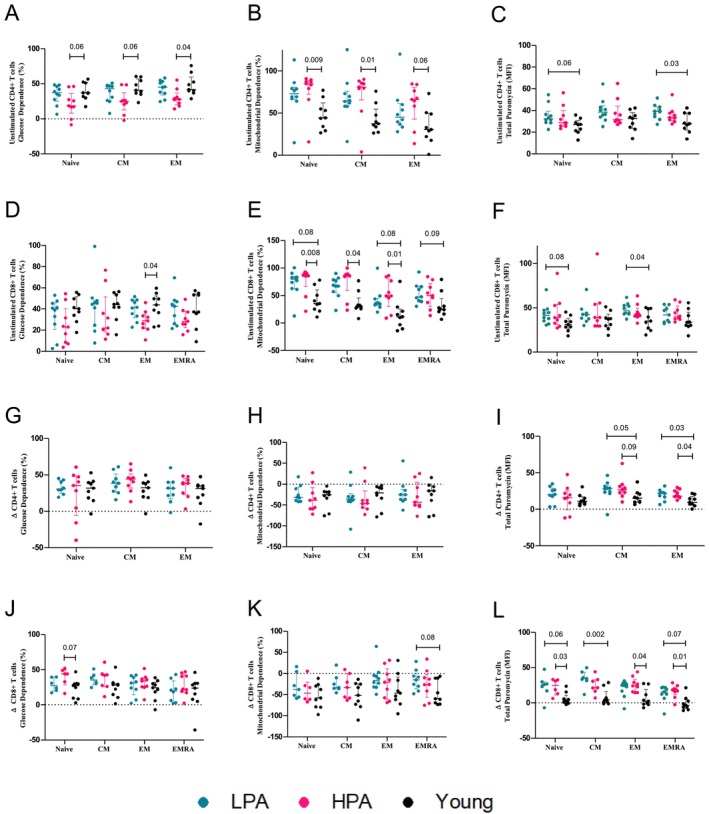
Effects of aging and PA on the metabolic profile of T cells. Comparison of glucose dependence (GD), mitochondrial dependence (MD) and protein synthesis (total puromycin incorporation) in unstimulated and stimulated CD4+ and CD8+ T cells isolated from younger adults and older adults with lower physical activity (LPA) and higher physical activity (HPA). Data for unstimulated CD4^+^ and CD8^+^ T cells are presented in figures (A–C) and (D–F) respectively. Data for stimulated (PMA and Ionomycin) CD4^+^ and CD8^+^ T cells are presented in figures (G–I) and (J–L) respectively. Data from stimulated cells are presented as delta values (Δ), which were calculated as the percentage of stimulated minus the percentage of unstimulated. Individual data points are presented and are accompanied by median and interquartile ranges (whiskers). *P*‐values from Independent‐Samples Mann–Whitney *U*‐tests adjusted by Bonferroni correction are presented, after confirming interaction via independent sample Kruskal–Wallis tests.

GD was significantly lower in unstimulated EM CD4^+^ and CD8^+^ cells of HPA older adults compared to young participants, and trended toward a lower level in naïve and CM CD4^+^ and CD8^+^ cells (Figure 1A,D). MD in unstimulated naïve, EM, and CM CD4^+^ and CD8^+^ cells of HPA older adults was significantly higher than young, and trended toward a higher level in naïve, EM, and EMRA CD8^+^ cells of LPA older adults compared to young (Figure 1B,E). Our analysis revealed overall protein synthesis was only higher in EM CD4^+^ and CD8^+^ of LPA adults, with a trend toward a higher level in CD4^+^ and CD8^+^ naïve T cell when compared to young (Figure 1C,F).

Taken together, these findings suggest that while T cells from older adults rely on a higher energy demand to sustain baseline functions, higher levels of PA prevented this increase. The underlying mechanisms of this higher energy demand in older adults' T cells remain to be determined in this cohort. Possible factors include chronic activation of the immune system, which occurs with aging (Franceschi et al. 2000), as well as increased mitochondrial dysfunction, reduced autophagy, and a higher prevalence of latent infections in older adults such as cytomegalovirus (Haynes 2020). The differences we report here in the metabolic profile of T cells with age show that aging increases MD, which could be due to impaired glycolysis. Indeed, previous studies have shown that aged T cells with dysfunctional mitochondria display impaired respiration, reduced glycolysis, and inefficient one‐carbon metabolism (Desdín‐Micó et al. 2020; Møller et al. 2022; Ron‐Harel et al. 2018). On the other hand, considering that PA led to even higher MD in CD4^+^ and CD8^+^ subsets, it is possible that PA partially prevents mitochondrial dysfunction and allows the cells to rely even more on this pathway. On this note, previous studies have shown that older individuals have increased mitochondrial mass and fusion, but also defective autophagy and increased influx and storage of lipids, which impair T‐cell proliferation and immune responses in older adults (Alsaleh et al. 2020; Bektas et al. 2019; Nicoli et al. 2022; Quinn et al. 2023).

Although higher MD in unstimulated T cells could be interpreted as a PA benefit, our results highlight the need for a deeper understanding of the causes and consequences of increased mitochondrial activity with age and PA. Higher mitochondrial activity at rest with aging may also be linked with the known increase in cytoplasmic mitochondrial DNA (mtDNA) that leads to hyperinflammatory responses and cell senescence (López‐Polo et al. 2024; Martini and Passos 2023). Although both LPA and HPA older groups increased MD, the potentially improved mitochondrial function with PA could prevent the need for the extra higher energy demand in this group (especially in effector memory T cell). Indeed, PA leads to higher mitochondrial respiration in T cells (Andonian et al. 2020; Gebhardt et al. 2024) via more efficient metabolism (e.g., upregulation of AMPK, higher ß‐oxidation and lower lipid accumulation) and potentially less oxidative damage (ROS production, mtDNA leakage, mitochondria quality control, autophagic capacity) (Callender et al. 2021; Liu et al. 2023; Moreira et al. 2017).

### 1.2 Metabolic Profile of Activated CD4 ^+^ and CD8 ^+^ T Cell

Upon activation, the increase in GD and reduction in MD in CD4^+^ subsets was similar across groups (Figure 1G,H), which could be due to the good health status of our older cohort. The overall increase in protein synthesis upon stimulation was higher in both LPA and HPA older adults for most CD4^+^ and CD8^+^ subsets, compared to young adults (Figure 1I,L).

The ability of a cell to respond or adapt to conditional changes in metabolic demand is known as metabolic flexibility. T cells of old mice and humans have a reduced metabolic flexibility, characterized by an impaired switch toward glycolytic metabolism upon activation compared to young individuals (Møller et al. 2022; Nian et al. 2021; Nicoli et al. 2022; Quinn et al. 2023; Ron‐Harel et al. 2018). The accumulation of glucose and lipids in CD8^+^ T cells contributes to a senescent phenotype, with increased p‐p53 expression and fragmented mitochondrial morphology, mirroring the features of EMRA CD8^+^ T cells of diabetic older adults (Callender et al. 2021). Therefore, it is possible that the sustained higher MD in older adults is associated with the accumulation of these intracellular substrates favoring T‐cell exhaustion (Yu et al. 2020).

Although not statistically significant, we observed a trend toward reduced MD upon activation in LPA EMRA CD8^+^ T cells compared to young, suggesting an impaired metabolic flexibility in this group; while HPA older adults demonstrated similar values to the young (Figure 1K). Additionally, there was a trend to higher GD in stimulated naïve CD8^+^ T cells in HPA older adults compared to young (Figure 1J). These confusing findings could be caused by a mix of compensatory effects that, on one hand, do not hold the increase in the energy demand in both older groups but potentially allow HPA to reduce MD in favor of a more efficient metabolic pathway (glycolysis) to sustain T‐cell responses upon activation. High heterogeneity, low sample size, and insufficiently different levels of physical activity between LPA and HPA in our study may have prevented the capture of greater differences in metabolic flexibility between these two groups. Exercise improves insulin sensitivity, glucose uptake, and lactate clearance in various cell types, and it is thus possible that it increases glycolytic efficiency (Smith et al. 2023). Glycolysis supports immune responses independent of ATP production, for example, by increasing intermediates that feed into biosynthetic pathways to facilitate proliferation, differentiation, and cytokine production, such as IFNy (Soto‐Heredero et al. 2020). The higher inflammatory phenotype in memory T cells of older adults occurs in parallel with higher respiratory capacity, mitochondrial content, and intracellular ROS production, without altering glucose uptake and cellular ATP levels (Chen et al. 2022). Nevertheless, how the impaired metabolic flexibility relates to energy demand and the inflammatory response in the context of exercise remains poorly understood.

**FIGURE 2:**
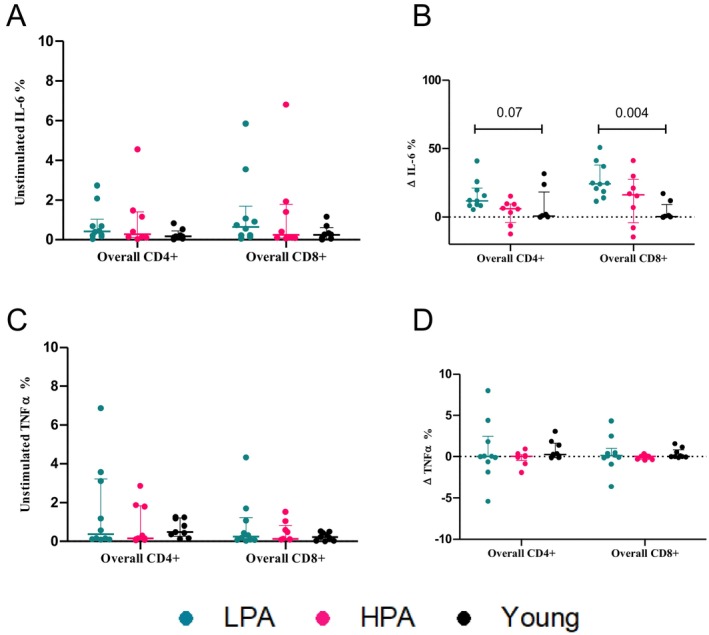
Effects of aging and PA on cytokine production by resting and stimulated CD4^+^ and CD8^+^ T cell. Comparison of interleukin(IL)‐6 and tumor necrosis factor‐alpha (TNF‐α) production by unstimulated and stimulated (PMA and ionomycin) CD4^+^ and CD8^+^ T cell isolated from younger adults and older adults with higher physical activity (HPA) and lower physical activity (LPA). Data for unstimulated cells are presented in (A, C) with data obtained from stimulated T cell presented in (B, D). Cytokine production by stimulated T cell is presented as delta values (Δ), which was calculated as stimulated % positive cells—unstimulated % positive cells. Individual data points are presented and are accompanied by median and interquartile range (whiskers). *P*‐values from independent sample Mann–Whitney *U*‐tests adjusted by Bonferroni correction are presented, after confirmed interaction via independent sample Kruskal–Wallis tests.

Upon activation, both older groups exhibited higher protein synthesis in multiple T‐cell subsets compared to young; only LPA adults had a significantly higher increase in the frequency of IL‐6 + CD8^+^ cells and trended toward a higher percentage of IL‐6 + CD4^+^ than the young counterparts (Figure 2B). No difference was observed for TNF‐α (Figure 2C,D), nor IL‐6 in unstimulated cells (Figure 2A). Although a robust increase in IL‐6 and TNF‐α is necessary for an efficient T‐cell immune response in healthy individuals (Dong 2021; Tanaka et al. 2014), aging often leads to exacerbated and detrimental cytokine production (Akbar et al. 2016; McNerlan et al. 2002; O'Mahony et al. 1998). For example, the ability of older adults to respond to antigenic stimulation is inversely correlated with their elevated inflammatory response (Jacinto et al. 2018) and the blockade of specific cytokines (i.e., TNF‐α) or systemic inflammation can restore antigenic responses (Puchta et al. 2016; Vukmanovic‐Stejic et al. 2018). Another explanation for the hyperinflammatory response with age is the potential higher frequency of senescent‐like T cells in LPA older adults (Freund et al. 2010). Although we did not specifically examine the metabolic profile of senescent T cells in this study, older adults had a higher frequency of CD8^+^ EMRAs, which encompass senescent‐like T cells (Callender et al. 2018) (S4). Determining the effect of PA on senescent T‐cell metabolism could explain how exercise improves immunosenescence and inflammaging. The reduced need for high levels of cytokine production in HPA could also be due to a higher density of cytokine receptors on T cells, as has been reported for IL‐6 receptors on muscle of physically trained individuals (Hunter and Jones 2015; Keller et al. 2005; Nash et al. 2023; Pedersen and Febbraio 2008). This in turn would hold the hyperinflammatory responses in HPA, ameliorating immunosenescence (Chambers and Akbar 2020). This could be particularly evident for IL‐6 since the increase in this cytokine upon in vitro stimulation is higher in individuals susceptible to cold or flu, contrary to the response of other cytokines, such as IFN**γ** (Meng et al. 2015). In an experimental autoimmune model, 6 weeks of training reduced IL‐6 concentration in vitro upon stimulation, in parallel with reduced clinical severity score (Zaychik et al. 2021). However, there is a clear scarcity of studies testing PA effects on cytokine production, particularly IL‐6 and TNF‐α, in activated T cells of older adults.

One limitation of this study is the relatively small differences in PA levels between our LPA and HPA cohorts. A possible explanation for these atypical levels is that this cohort was initially required to be disease‐free at the time of recruitment, and it is no surprise that maintaining good health at seventies requires a certain level of PA. Therefore, the magnitude of PA's effects on T‐cell metabolism may be greater when compared to nonphysically active groups, which are more prevalent among older adults (NHS digital 2023). Missing data was another limitation for certain T‐cell subsets. SCENITH does require a consistent cell count across experimental conditions, which particularly impacts populations with low cell counts such as EMRA CD4^+^ T cells (excluded from analysis).

In summary, our findings suggest a higher energy demand, impaired metabolic flexibility, and hyperinflammatory responses in aged T cells. PA reduces the metabolic demand in these cells via a compensatory increase in MD and improved metabolic flexibility. This could partially explain the benefits of PA in delaying immunosenescence. It highlights the need for future research to identify the cellular regulators of T‐cell fitness that are influenced by PA to develop new means of preventing and treating immunosenescence in older adults.

## Author Contributions

A.V.S. conceived the study, secured funding, and managed overall direction. A.V.S. and J.H. designed experiments. A.V.S. and E.W. collected samples. E.W. performed all experiments. E.W., J.H., and A.V.S. analyzed the data and wrote the manuscript. N.A.D., A.L., and J.M.L. critically revised the manuscript. All authors approved the final version of the manuscript.

## Conflicts of Interest

The authors declare no conflicts of interest.

## Supporting information


Appendix S1.

Appendix S2.

Appendix S3.

Appendix S4.

Appendix S5.

## Data Availability

The data that support the findings of this study are available on request from the corresponding author. The data are not publicly available due to privacy or ethical restrictions.
