# Supplementary material for: Physical Activity Modifies the Metabolic Profile of CD4 + and CD8 + T‐Cell Subtypes at Rest and Upon Activation in Older Adults
Source: Aging Cell. 2025 May 21;24(7):e70104. doi: 10.1111/acel.70104 (PMC12266771; doi:10.1111/acel.70104)
Supplement: Supplementary file 1 — Appendix S1. [file ACEL-24-e70104-s005.docx]

**S1. Methods.**

***Population.*** Ethical approval for this study was granted by the HRA and Health and Care Research Wales (HRCW) Approval (IRAS 301974) and written consent was obtained from individuals prior to participation. Older participants had to be >65 years of age and younger participants had to be between 18-35 years of age. All participants were required to be in good health at the time of the study, which was defined as without infection, disease or taking immunosuppressive medication (per the criteria set out by Greig *et al* (Greig et al., 1994)). Participants completed a health questionnaire, a physical functioning assessment and donated blood samples. The physical functioning assessment consisted of hand grip strength, walking gait speed, and 30-second sit-to-stand tests. Blood samples were collected into heparinized vacutainers® (BD Biosciences, New Jersey, USA).

Peripheral blood mononuclear cell (***PBMC) isolation.*** The collected blood samples were pooled from heparinised vacutainers into 25 ml universal tubes (Scientific Laboratory Supplies, Nottingham, UK) and diluted with RPMI-1640 media supplemented with glutamine, penicillin and streptomycin (Life Technologies Limited, Paisley, UK) at a 1:1 ratio. The blood-RPMI mix was layered on top of 6 ml of Ficoll-Plaque^TM^ Plus (GE Healthcare, Buckinghamshire, UK) and centrifuged at 400 × g for 30 minutes at room temperature (RT) with no break or acceleration. After centrifugation, PBMCs were transferred into a fresh universal tube containing MACS running buffer (Miltenyi Biotec, Surrey, UK). Once all PBMCs were transferred, the universal tube was topped up with autoMACS running buffer and centrifuged at 300 × g for 10 mins at RT with full brake and acceleration. Post spin, the pellet was resuspended in 25ml autoMACS running buffer and washed via centrifugation at 300 × g for 10 minutes at RT. 1 ml of a prepared freezing solution containing 5 ml 10% DMSO (Sigma Alrich, Dorset, UK) and 45 ml heat-inactivated FCS (Thermofisher, Massachusetts, USA) was used to resuspend the pellet. 500 µl of PBMCs were aliquoted into 1 ml cryovials (Greiner Bio-One, Gloucestershire, UK) and then placed in a Mr Frosty^TM^ Freezing Container (ThermoFisher, Massachusetts, USA) in which they were frozen at -80°C.

***PBMC stimulation.*** PBMCs were removed from the freezer, thawed at 37°C and added to a universal tube containing 10 ml of warm RPMI-1640 media with no penicillin and streptomycin (P/S). This PBMC-RMPI medium mix was centrifuged at 300 × g for 10 minutes at RT, resuspended in 1 ml of warm RMPI medium with no P/S, and incubated for 15 hours at 37°C, 5% CO_2_. Post incubation, 9 ml of warm RPMI-1640 medium with no P/S was added to the PBMCs and the mix was centrifuged at 300 × g for 10 minutes at RT and resuspended in 1 ml of warm RPMI medium with no P/S. One hundred microliters of this mix were added to a 1.5 ml microtube (Sarstedt AG & Co., Nümbrecht, Germany) and the white blood cell (WBC) count was determined using a Sysmex XN-1000 analyzer (Sysmex, Milton Keynes, UK). The PBMC mixture was diluted with a warm RPMI medium with no P/S to a concentration of 2×10^6^ /ml. 100 µl of the PBMC mixture was aliquoted into 5 ml polypropylene round-bottom tubes (Scientific Laboratory Supplies, Nottingham, UK), stimulated with 2µl Phorbol 12-myristate 13-acetate (PMA) (50 ng/ml; Merck Life Science, Dorset, UK) and 2 µl Ionomycin (500 ng/ml; Merck Life Science, Dorset, UK), and incubated at 37°C, 5% CO_2,_ for 4 hours. Untreated PBMCs were used as negative controls.

***T cell metabolic profile.*** The flow cytometry method, single cell energetic metabolism by profiling translation inhibition (SCENITH) (Argüello et al., 2020), was used to investigate the metabolic profiles of CD4^+^ and CD8^+^ T cell subtypes. Almost half of the energy produced by mammalian cells via metabolic processes is used up by protein synthesis machinery (Lindqvist et al., 2018). The incorporation of puromycin is a widely accepted readout for protein synthesis *in vitro* and *in vivo* (Aviner, 2020; Hidalgo San Jose & Signer, 2019; Seedhom et al., 2016). Therefore, using an anti-puromycin monoclonal antibody, the metabolic activity of cells can be measured by puromycin levels via flow cytometry. Metabolic inhibitors are utilized to determine the metabolic profile of a cell, defined by glucose and mitochondria dependence. 2-Deoxy-D-Glucose (2DG) inhibits glycolysis, and therefore, it allows for the calculation of glucose dependence (Singh et al., 2023), whilst oligomycin inhibits oxidative phosphorylation, allowing for the calculation of mitochondria dependence (Mackieh et al., 2023).

Thawed PBMCs were incubated for 15 hours (37 °C, 5%CO_2_) prior to the experiment. Immediately after being stimulated or left untreated as the control, PBMCs were treated with either 10 µl of 2DG (100 mM; Merck Life Science, Dorset, UK), 1 µl Oligomycin from *Streptomyces diastatochromogenes* (1 µM; Merck Life Science, Dorset, UK]), a combination of the drugs at the same concentrations, or left untreated as the control, and incubated at 37°C, 5% CO_2_ for 15 minutes. Following incubation, PBMCs were treated with 5 µl Puromycin dihydrochloride from *Streptomyces alboniger* (10 µg/ml; Merck Life Science, Dorset, UK) and left to incubate at 37°C, 5% CO_2_ for a further 30 minutes. PBMCs were then washed with 200 µl phosphate buffered saline (PBS) via centrifugation at 250 × g for 5 minutes at 4°C and stained with surface marker conjugated antibodies CD3 (PE-Cyanine7; Life Technologies Corp, California, USA), CD4 (Brilliant Violet 421^TM^; BioLegend®, California, USA), CD8 (PE; BioLegend®, California, USA), CCR7 (APC; BioLegend®, California, USA), and CD45RA (PerCP; BioLegend®, California, USA) and left to incubate on ice for 20 minutes protected from light. Post incubation, PBMCs were washed with 300 µl PBS at 250 × g for 5 minutes at 4°C and then fixed and permeabilised (FOXP3 transcription factor Staining Buffer Set; Fisher Scientific, Leicestershire, UK), stained with Human TruStain FcX^TM^ (BioLegend®, California, USA) to prevent any non-specific binding to the Fc region of the antibodies. After 5 minutes, anti-Puromycin (Alexa Fluor® 488 anti-puromycin; BioLegend®, California, USA) or isotype control (Alexa Fluor® 488) was added, and incubated for 1-hour at 4°C without any light. After staining, PBMCs were washed with 200 µl PBS at 250 × g for 5 minutes at 4°C and resuspended in 200 µl PBS. Samples were kept on ice and protected from light prior to analysis.

***T cell cytokine production.*** After a 4-hour incubation period with Brefeldin A (10 µg/ml; Merck Life Science, Dorset, UK) and with or without stimulation, PBMCs were washed with 300 µl PBS via centrifugation at 250 × g for 5 minutes at 4°C. Once resuspended in 50 µl PBS, PBMCs were stained with surface marker conjugated antibodies CD3 (PE-Cyanine7; Life Technologies Corp, California, USA), CD4 (Brilliant Violet 421^TM^; BioLegend®, California, USA), CD8 (VioGreen^TM^; Miltenyi Biotec, Surrey, UK), CCR7 (APC; BioLegend®, California, USA), and CD45RA (PerCP; BioLegend®, California, USA) and left to incubate in ice protected from light for 20 minutes. Post-incubation, PBMCs were washed with 300 µl PBS at 250 × g for 5 minutes at 4°C and fixed via a 30-minute incubation with 50 µl Medium A (Life Technologies LTD, Paisley, UK) at RT. PBMCs were then washed via centrifugation at 250 × g for 5 minutes at 4°C and resuspended in 50 µl Medium B for permeabilization. PBMCs underwent intracellular staining for IL-6 (PE; BioLegend®, California, USA) and TNFα (FTIC; BD Pharmingen, California, USA) and were then left to incubate on ice for 30 minutes without light. After incubation, PBMCs were washed with 300 µl PBS at 250 × g for 5 minutes at 4°C and resuspended in 200 µl PBS. Samples were kept on ice and protected from light prior to analysis.

***Flow cytometry.*** For SCENITH analysis, the distribution of CD4^+^ and CD8^+^ T cells subsets were determined. Each T cell subset was defined as CD4^+^ naïve (CD3^+^CD4^+^CD45RA^+^CCR7^+^), CD4^+^ central memory (CM, CD3^+^CD4^+^CD45RA^-^CCR7^+^), CD4^+^ effector memory (EM, CD3^+^CD4^+^CD45RA^-^CCR7^-^), CD4^+^ terminally differentiated effector memory cells (CD3^+^CD4^+^CD45RA^+^CCR7^-^), CD8^+^ naïve (CD3^+^CD8^+^CD45RA^+^CCR7^+^), CD8^+^ central memory (CM, CD3^+^CD8^+^CD45RA^-^CCR7^+^), CD8^+^ effector memory (EM, CD3^+^CD8^+^CD45RA^-^CCR7^-^) or CD8^+^ terminally differentiated effector memory cells (CD3^+^CD8^+^CD45RA^+^CCR7^-^) (S2 a-d). The metabolic profile of each T cell subset was quantified by the median fluorescence intensity (MFI) of puromycin post treatment with metabolic inhibitors (2DG and Oligomycin) or isotype control (S2 e and f). Metabolic profile was defined by glucose and mitochondria dependence, and we also considered maximum protein synthesis as Puromycin MFI (without inhibition) Glucose and mitochondria dependence were determined by the calculations provided:

$$Glucose dependence=\frac{100(PuroMFI-PuroMFI with 2DG inhibition)}{(PuroMFI-PuroMFI with 2DG and Oligomycin inhibition)}$$

$$Mitochondria dependence=\frac{100(PuroMFI-PuroMFI with Oligomycin inhibiton)}{(PuroMFI-PuroMFI with 2DG and Oligomycin inhibition}$$

Importantly, when puromycin MFI were as lower as the isotype control (below 4), which was usually caused by the population of cells with low counts (< 300 events), the data was excluded for analysis. For the T cell cytokine experiments, CD4^+^ and CD8^+^ T cell populations were determined. These populations were defined by: CD4^+^ (CD3^+^CD4^+^CD8^-^), CD8^+^ (CD3^+^CD4^-^CD8^+^) (S3 a-b). To assess cytokine production of the CD4^+^ and CD8^+^ T cell populations, TNFα and IL-6 MFI and % were measured via anti-TNF and anti-IL-6 (S3 c-f).

All flow cytometry was performed on a MACSQuant® Analyzer 8 flow cytometer (Miltenyi Biotec. Surrey, UK) and analyzed by FlowJo software (FlowJo LLC, Orlando, USA). The key resources are listed below (S5).

***Statistical analysis.*** Statistical analyses were performed using IBM SPSS software version 29.0 (IBM, Portsmouth, UK). The normality of the data was assessed using the Kolmogorov-Smirnov test. The majority of variables were non-normally distributed, and therefore the comparison across the 3 groups were tested by Independent-Samples Kruskal-Wallis test, followed by Independent-Samples Mann-Whitney U tests adjusted by Bonferroni correction for multiple tests. Differences were considered statistically significant at p-value ≤ 0.05, and the tendency to significant differences (p-value from 0.05 to 0.1) were also reported. Results were presented using GraphPad PRISM® software (GraphPad software, California, USA). Individual data with median and interquartile range are presented in the graphs, whilst baseline characteristics are presented as mean ± standard deviation.

**References**

Argüello RJ, Combes AJ, Char R, Gigan J-P, Baaziz AI, Bousiquot E, Camosseto V, Samad B, Tsui J, Yan P, Boissonneau S, Figarella-Branger D, Gatti E, Tabouret E, Krummel MF & Pierre P (2020). SCENITH: A Flow Cytometry-Based Method to Functionally Profile Energy Metabolism with Single-Cell Resolution. *Cell Metab* 32, 1063-1075.e7. https://www.scopus.com/inward/record.uri?eid=2-s2.0-85096689445&doi=10.1016%2Fj.cmet.2020.11.007&partnerID=40&md5=688ef21d309a13019f5f148f0eba963a.

Aviner R (2020). The science of puromycin: From studies of ribosome function to applications in biotechnology. *Comput Struct Biotechnol J* 18, 1074–1083. https://pubmed.ncbi.nlm.nih.gov/32435426/.

Greig CA, Young A, Skelton DA, Pippet E, Butler FMM & Mahmud SM (1994). Exercise studies with elderly volunteers. *Age Ageing* 23, 185–189. https://pubmed.ncbi.nlm.nih.gov/8085501/.

Hidalgo San Jose L & Signer RAJ (2019). Cell-type-specific quantification of protein synthesis in vivo. *Nat Protoc* 14, 441–460. https://pubmed.ncbi.nlm.nih.gov/30610239/.

Lindqvist LM, Tandoc K, Topisirovic I & Furic L (2018). Cross-talk between protein synthesis, energy metabolism and autophagy in cancer. *Curr Opin Genet Dev* 48, 104–111. https://pubmed.ncbi.nlm.nih.gov/29179096/.

Mackieh R, Al-Bakkar N, Kfoury M, Roufayel R, Sabatier JM & Fajloun Z (2023). Inhibitors of ATP Synthase as New Antibacterial Candidates. *Antibiot (Basel, Switzerland)* 12. https://pubmed.ncbi.nlm.nih.gov/37107012/.

Seedhom MO, Hickman HD, Wei J, David A & Yewdell JW (2016). Protein Translation Activity: A New Measure of Host Immune Cell Activation. *J Immunol* 197, 1498–1506. https://pubmed.ncbi.nlm.nih.gov/27385780/.

Singh R, Gupta V, Kumar A & Singh K (2023). 2-Deoxy-D-Glucose: A Novel Pharmacological Agent for Killing Hypoxic Tumor Cells, Oxygen Dependence-Lowering in Covid-19, and Other Pharmacological Activities. *Adv Pharmacol Pharm Sci* 2023. https://pubmed.ncbi.nlm.nih.gov/36911357/.
