# Supplementary material for: Physical Activity Modifies the Metabolic Profile of CD4 + and CD8 + T‐Cell Subtypes at Rest and Upon Activation in Older Adults
Source: Aging Cell. 2025 May 21;24(7):e70104. doi: 10.1111/acel.70104 (PMC12266771; doi:10.1111/acel.70104)
Supplement: Supplementary file 2 — Appendix S2. [file ACEL-24-e70104-s002.docx]

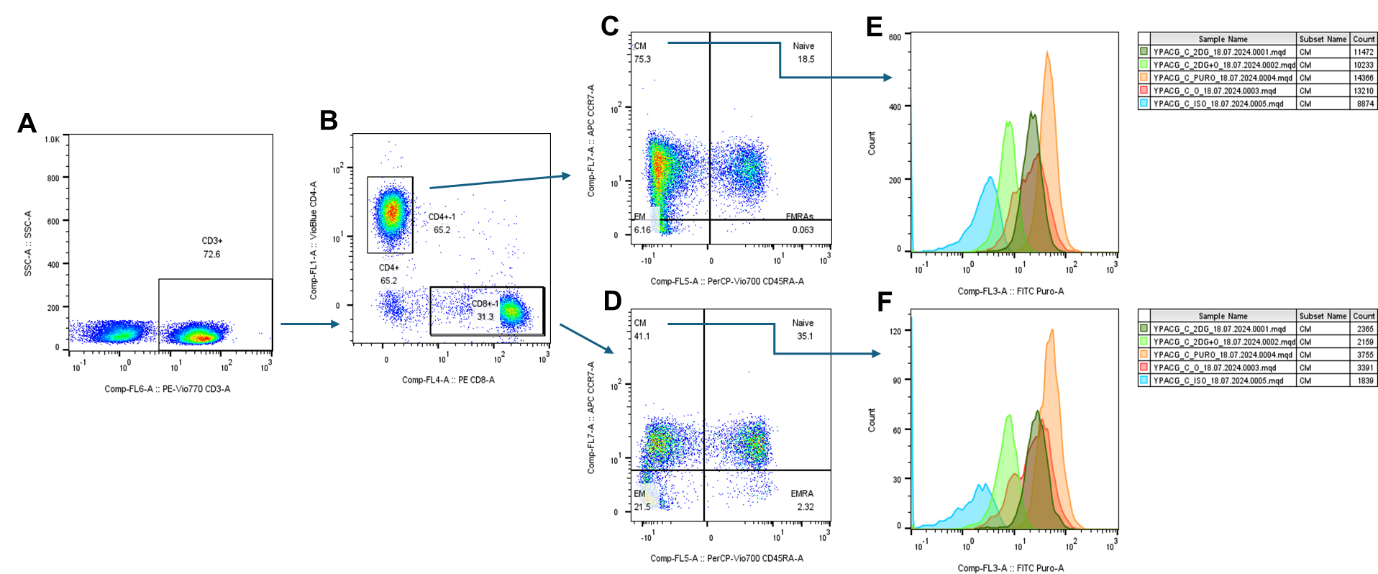


**S2. Gating strategy for SCENITH protocol.** PBMCs were stained with anti-CD3 (A), anti-CD4 and anti-CD8 (B), anti-CD-45RA and anti-CCR7 (C and D), and Alexa Fluor 488 anti-puromycin and Alexa Fluor 488 Mouse IgG2a k isotype control (E and F).
