# Supplementary material for: Physical Activity Modifies the Metabolic Profile of CD4 + and CD8 + T‐Cell Subtypes at Rest and Upon Activation in Older Adults
Source: Aging Cell. 2025 May 21;24(7):e70104. doi: 10.1111/acel.70104 (PMC12266771; doi:10.1111/acel.70104)
Supplement: Supplementary file 3 — Appendix S3. [file ACEL-24-e70104-s004.docx]

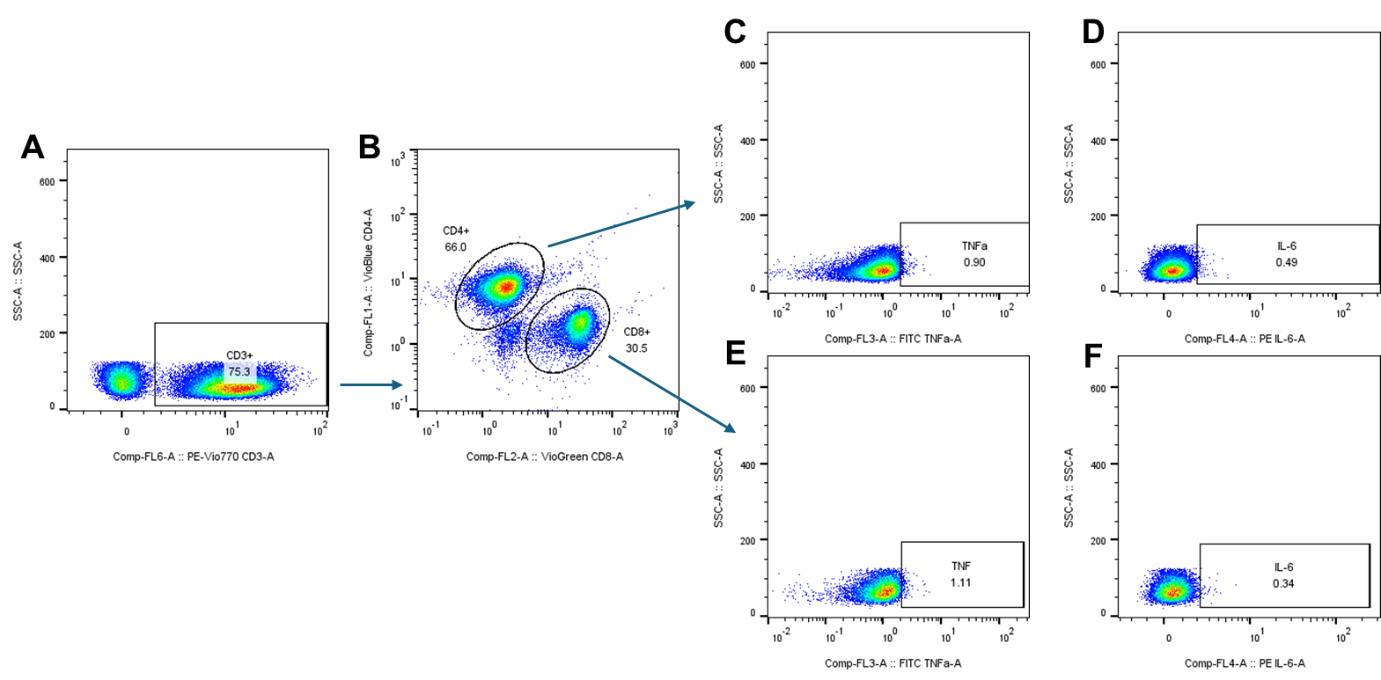


**S3. Gating strategy for T cell cytokine production.** PBMCs were stained with anti-CD3 (A), anti-CD4 and anti-CD8 (B), and anti-TNFα and anti-IL-6 antibodies (C-F).
