# Supplementary material for: Physical Activity Modifies the Metabolic Profile of CD4 + and CD8 + T‐Cell Subtypes at Rest and Upon Activation in Older Adults
Source: Aging Cell. 2025 May 21;24(7):e70104. doi: 10.1111/acel.70104 (PMC12266771; doi:10.1111/acel.70104)
Supplement: Supplementary file 4 — Appendix S4. [file ACEL-24-e70104-s003.docx]

| **S4.** Participant characteristics and immune phenotype. | | | |
| --- | --- | --- | --- |
| ***Characteristics*** | **Young (n=9)** | **Old HPA (n=9)** | **Old LPA (n=10)** |
| Age (years) | **23.7 ± 3.2^bc^** | **75.6 ± 4.3^a^** | **76 ± 2^a^** |
| Sex | m=7, f=2 | m=4, f=5 | m=4, m=6 |
| Weight (kg) | 73.8 ± 16.9 | 66.4 ± 10.7 | 72.6 ± 20.1 |
| BMI (kg/m^2^) | 22.5 ± 3.4 | 24 ± 3 | 26.9 ± 5.1 |
| ***Physical activity and physical fitness*** |  |  |  |
| Mild (hours /week) | **5.7 ± 1.6^b^** | **9.6 ± 2.5^ac^** | **5.6 ± 3.8^b^** |
| Moderate (hours /week) | 2.6 ± 2.3 | 2.3 ± 1.5 | 0.7 ± 1.4 |
| Vigorous (hours/week) | **4.3 ± 2.8^c^** | 1.4 ± 1.7 | **0.9 ± 2.5^a^** |
| Sedentary time (hours/d) | 1.3 ± 1.3 | 0.7 ± 0.8 | 1.1 ± 1 |
| Grip Strength (kg) | **37.1 ± 9.8^c^** | 26.8 ± 6.6 | **23.6 ± 6.5^a^** |
| Gait Speed (m/s) | 1.3 ± 0.3 | 1.3 ± 0.3 | 1.2 ± 0.4 |
| 30-second sit-to-stand test (reps) | **19.8 ± 5^c^** | 13.9 ± 3.7 | **13.1 ± 4.4^a^** |
| **Immune phenotype** |  |  |  |
| T CD4+ % | **59.2 ± 9.2^c^** | 69 ± 0.2 | **75.4 ± 13.8^a^** |
| T CD4+ naïve % | 46.9 ± 16.5 | 40.8 ± 14.9 | 36.2 ± 19.1 |
| T CD4+ Central memory % | 40.1 ± 14.8 | 50.1 ± 11.7 | 51.1 ± 21.4 |
| T CD4+ Effector memory % | 12.1 ± 6.8 | 8.5 ± 5.1 | 9.7 ± 10.4 |
| T CD4+ EMRA % | 0.8 ± 0.9 | 0.6 ± 0.3 | 3 ± 6.3 |
| T CD8+ % | **34.5 ± 7.4^c^** | 26.5 ± 15.3 | **20 ± 12.1^a^** |
| T CD8+ naïve % | **45.1 ± 16.7^c^** | 19.4 ± 11.3 | **17.3 ± 14.4^a^** |
| T CD8+ Central memory % | 18.8 ± 15.8 | 6.5 ± 3.8 | 8.9 ± 7.7 |
| T CD8+ Effector memory % | **26.8 ± 13.8^bc^** | **52.5 ± 12.9^a^** | **51 ± 19.2^a^** |
| T CD8+ EMRA % | **9.4 ± 7.9^b^** | **21.7 ± 8.1^a^** | 22.8 ± 20.5 |

Groups were defined by age and moderate-to-vigorous activity (MVPA). Participant characteristics were determined via a health questionnaire. The physical activity of participants was quantified via a health questionnaire and physical functioning assessment, consisting of hand grip strength, walking gait speed, and a 30-second sit-to-stand test. The immune phenotype of participants was defined by PBMCs isolated from blood samples and assessed via flow cytometry. T cell subsets were defined as CD4^+^ (CD3^+^CD4^+^), CD4^+^ naïve (CD3^+^CD4^+^CD45RA^+^CCR7^+^), CD4^+^ central memory (CD3^+^CD4^+^CD45RA^-^CCR7^+^), CD4^+^ effector memory (CD3^+^CD4^+^CD45RA^-^CCR7^-^), CD4^+^ EMRA, terminally differentiated effector memory (CD3^+^CD4^+^CD45RA^+^CCR7^-^), CD8^+^ (CD3^+^CD8^+^), CD8^+^ naive (CD3^+^CD8^+^CD45RA^+^CCR7^+^), CD8^+^ central memory (CD3^+^CD8^+^CD45RA^-^CCR7^+^), CD8^+^ effector memory (CD3^+^CD8^+^CD45RA^-^CCR7^-^), CD8^+^EMRA terminally differentiated effector memory (CD3^+^CD4^+^CD45RA^+^CCR7^-^). LPA: lower physical activity, HPA: higher physical activity, a: significantly different from young, b: significantly different from old HPA, c: significantly different from old LPA. Significant differences were considered p< 0.05. Significance was tested by the Independent-Samples Kruskal-Wallis test. Data are presented as mean ± standard deviation (SD).
