# Supplementary material for: Physical Activity Modifies the Metabolic Profile of CD4 + and CD8 + T‐Cell Subtypes at Rest and Upon Activation in Older Adults
Source: Aging Cell. 2025 May 21;24(7):e70104. doi: 10.1111/acel.70104 (PMC12266771; doi:10.1111/acel.70104)
Supplement: Supplementary file 5 — Appendix S5. [file ACEL-24-e70104-s001.docx]

**S5.** List of key materials.

| **Reagent or Resource** | **Source** | **Identifier** |
| --- | --- | --- |
| ***Antibodies*** | | |
| Alexa Fluor 488 anti-puromycin (Clone: 2A4) | Biolegend | Cat#381506 |
| Alexa Fluor 488 Mouse IgG2a, k isotype Ctrl (Clone: MOPC-173) | Biolegend | Cat#400233 |
| Anti-human CD3 PE Cyanine-7 (Clone: UCHT1) | eBioscience | Cat#25-0038-42 |
| Anti-human CD4 Brilliant Violet 421 (Clone: RPA-T4) | Biolegend | Cat#300532 |
| Anti-human CD8 PE (Clone: QA18A37) | Biolegend | Cat#303804 |
| Anti-human CD8 VioGreen REAfinity (Clone: REA734) | Miltenyi Biotec | Cat#130-110-684 |
| Anti-human CD45RA PerCP (Clone: HI100) | Biolegend | Cat#304156 |
| Anti-human CD197 (CCR7) (Clone: G043H7) | Biolegend | Cat#353214 |
| Anti-human IL-6 PE (Clone: MQ2-13A5) | Biolegend | Cat#501107 |
| Anti-human TNF FITC | BD Pharmingen | Cat#552889 |
| Human TruStain FcX | Biolegend | Cat#422302 |
| ***Chemicals, peptides, and recombinant proteins*** | | |
| autoMACS Running Buffer | Miltenyi Biotec | Cat#130-091-221 |
| Brefeldin A | Merck | Cat#500583 |
| Dimethylsulfoxide (DMSO) | Sigma-Aldrich | Cat#D1435 |
| Ficoll-Paque PLUS | GE Healthcare | Cat#17-1440-03 |
| Fix & Perm Medium A | Invitrogen | Cat#GAS001S100 |
| Fix & Perm Medium B | Invitrogen | Cat#GAS002S100 |
| Heat-inactivated FCS | ThermoFisher | Cat#A3840001 |
| Ionomycin | Merck | Cat#407951 |
| MACSQaunt Running Buffer Concentrate (16x) | Miltenyi Biotec | Cat#130-111-562 |
| Oligomycin from *Streptomyces diastatochromogenes* | Merck | Cat#495455 |
| Penicillin and Streptomycin | ThermoFisher | Cat#15140122 |
| Phorbel 12-myristate 13-acetate (PMA) | Merck | Cat#524400 |
| Puromycin dihydrochloride from S*treptomyces alboniger* | Merck | Cat#P7255 |
| RPMI Medium 1640 (1X) | Life Technologies | Cat#21875-091 |
| 2-Deoxy-D-Glucose (2DG) | Merck | Cat#25972 |
| 200mM L-Glutamine | ThermoFisher | Cat#25030081 |
| ***Critical commercial assays*** | | |
| FOXP3/Transcription Factor Staining Buffer Set | eBioscience | Cat#00-5523-00 |
| ***Software*** | | |
| FlowJo | FlowJo | v10.10.0 |
| Prism 10 | Graphpad | V8.3 |
| SPSS | IBM | V 29.0 |
